# The Pathology, Diagnosis, and Treatment of Absolute Hysterical Deafness in Soldiers

**Published:** 1918

**Authors:** 


**The Pathology, Diagnosis, and Treatment of Absolute Hysterical Deafness in Soldiers.** By A. F. HURST, Temp. Major, R. A. M. C., and E. A. PETERS, Temp. Capt. R. A. M. C. Abs. from *The Lancet*, Oct. 6, 1917.

Hurst and Peters report two cases of complete hysterical deafness which had persisted for months in spite of treatment by the usual methods of suggestion, electricity, and efforts at reeducation. The deafness continued during natural sleep, since it was impossible to awaken the patients by loud noises; and even when hypnotized no sounds made any impression on them. All the usual tests for malingering were tried without any evidence that a sound could be heard, yet there were not the organic changes usually found in complete deafness. All the usual methods of treating hysterical deafness having failed, they were told that an operation on the ear would certainly cure them. A little ether was given and two incisions made behind the ears, an iron plate being continuously hammered at the same time making a loud noise near the ear. The reaction was the same with both patients — each jumped from the table when coming out from under the influence of the ether. Normal hearing returned to both in a few minutes after " operation ", and though months have elapsed the deafness has not returned and they have taken up their work in France.

After the report of the two cases the authors discuss the etiology, diagnosis, pathogenesis and treatment of hysterical deafness. Regarding etiology and pathogenesis they say :

The momentary deafness which is the natural result of the terrific noise caused by the explosion of a big shell in the immediate neighbourhood may make such an impression on the mind of a soldier that on coming to himself, whether he has actually lost consciousness or not, his first thought is for his hearing, especially if it was already impaired by preceding disease, and he may be so convinced that he is permanently deafened that he becomes actually deaf as a result of auto-suggestion. The temporary deafness which occurs in gunners from the effect of the constant repetition of very loud noises may in the same way become perpetuated and exaggerated by auto-suggestion. Organic deafness, especially if the onset is acute, as in that due to involvement of the auditory nerve trunk in cerebro-spinal fever, may remain complete after the disappearance of the active inflammation has been followed by restoration of some of the nerve fibres, so that a certain amount of hearing should have returned. This, again, is due to auto-suggestion, the final deafness being organic with a superadded hysterical element, which is capable of removal, like all hysterical symptoms, by suggestion.

Hearing necessitates listening. Inattention during a dull sermon results in total deafness to the sermon, and in hysterical deafness the patient is so convinced that he cannot hear that he does not listen. Although the sound vibrations reach the ear in the normal way, they do not give rise to the slightest auditory sensation because of this inattention. The synapses at one or more of the cell stations in the auditory path to the cerebral cortex must, therefore, be unswitched, probably as a result of retraction of the dendrons. In absolute hysterical deafness the auditory motor reflex, which is a function of the mid-brain, may be abolished or greatly diminished. One of the unswitched synapses must, therefore, be below the mid-brain and either in the auditory nucleus or else probably in one of the intermediate cell stations — the superior olive or the nucleus of the lateral fillet. The persistence of the deafness during hypnosis and natural sleep shows that during the prolonged inattention of hysterical deafness which has lasted for a considerable period the unswitching of the synapses is more profound than that which normally occurs during deep sleep, in which the synapses can always be forced by a loud noise.

The diagnosis is made from (1) the history; (2) quantitative tests for air and bone conduction — the watch test for air and the modified Schwabach test for bone conduction; (3) auditory motor, or jump, reflex; (4) persistence during sleep; (5) effect of hypnosis; (6) changes in the voice; and (7) the vestibular reaction.

They state that uncomplicated deafness is the most difficult of all hysterical symptoms to treat; mutism is one of the easiest. It is therefore fortunate that most cases of hysterical deafness are associated with mutism, as the return of speech in a deaf mute generally convinces him that he can also hear, especially if he has been told that this will be the case. He listens as soon as he finds he can speak, and the unswitched synapses are once more switched on.

Their methods of treatment consist of suggestion with the aid of cold or hot baths. The majority of cases of hysterical deaf-mutism were cured by the introduction of an intralaryngeal electrode without switching on the current, informing the patient that the application of electricity to the vocal cords will surely restore speech. Many hysterical deaf-mutes will talk when coming from under the influence of ether. After speech has returned it is then easy to persuade the patient that he can hear. The results from the two operations give encouragement for the cure of the cases of uncomplicated hysterical deafness that hitherto have resisted the usual methods of treatment.

